# Transmural gradients of preferential diffusion motility in the normal rat myocardium characterized by diffusion tensor imaging

**DOI:** 10.1186/1532-429X-17-S1-Q117

**Published:** 2015-02-03

**Authors:** Archontis Giannakidis, Pedro Ferreira, Grant T Gullberg, David Firmin, Dudley J Pennell

**Affiliations:** 1 NIHR Cardiovascular Biomedical Research Unit, Royal Brompton Hospital, London, UK; 2 National Heart and Lung Institute, Imperial College London, London, UK; 3 Life Sciences Division, Lawrence Berkeley National Laboratory, Berkeley, CA, USA; 4 Radiology and Biomedical Imaging, University California San Francisco, San Francisco, CA, USA

## Background

In recent years, diffusion tensor imaging (DTI) has emerged as a powerful noninvasive tool that infers the cardiac tissue organization through measuring water molecule motility.[1] Previous DTI studies [2] on normal hearts largely assumed that the measured diffusion magnitude along the 3 preferred directions and the measured degree of diffusion anisotropy remain the same over the endo-, meso-, and epi-cardial layers of the left ventricular (LV) wall. Our goal is to test the validity of this assumption.

## Methods

### MRI

DTI of 6 excised Wistar Kyoto rat hearts was performed on a 7T Bruker BioSpec using a 3D spin echo sequence with 12 optimized [3] diffusion directions at b=1000smm-2 (+b0) with 160μm isotropic resolution.

### Data analysis

**Figure 1:**
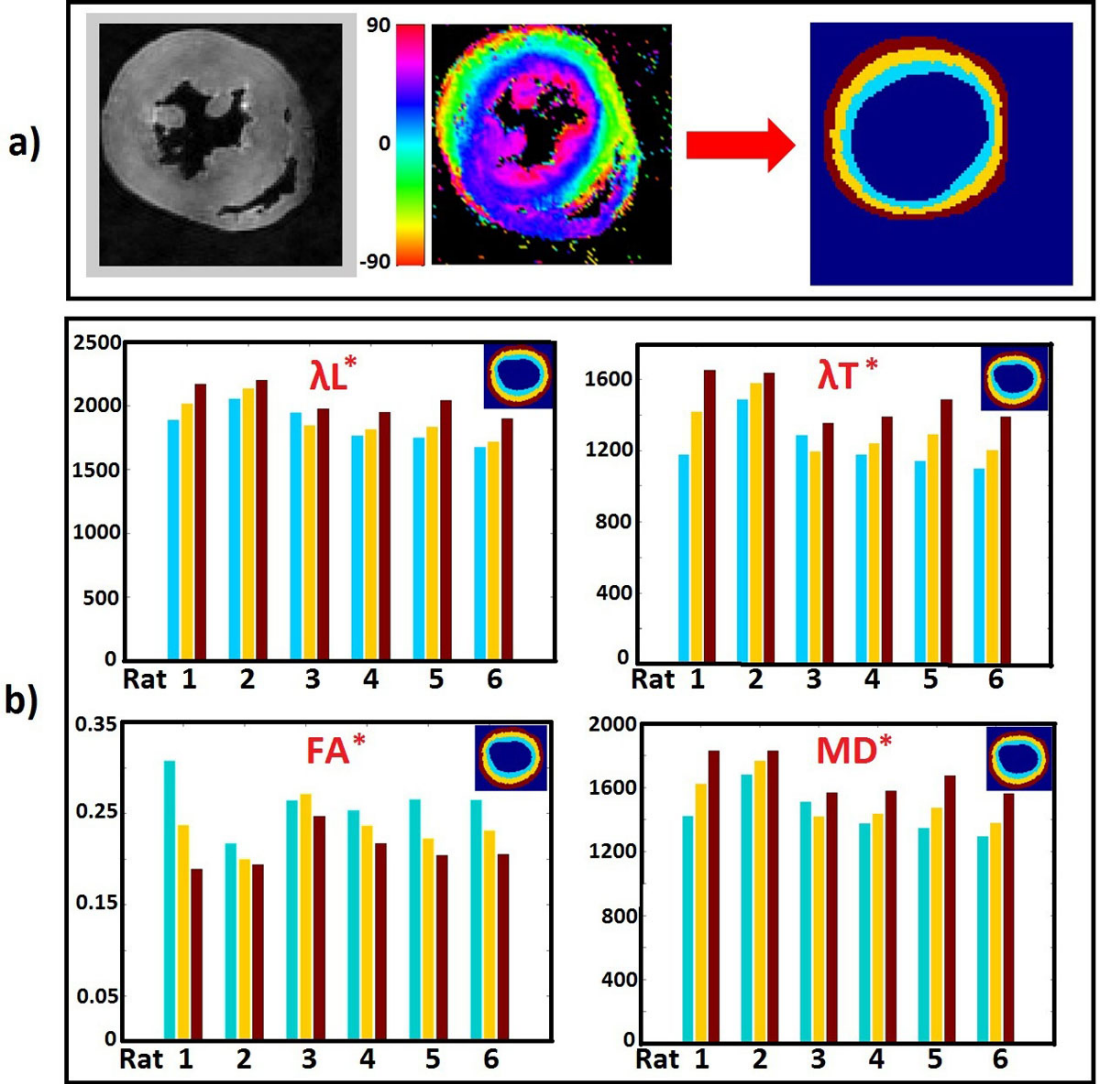
a) Determination of the region of interest. The equatorial short-axis slice with the largest cross-sectional area was analysed. The LV wall was delineated manually in the B0 data-set (left image). Extra care was taken to exclude papillary muscles and areas of signal dropout. To this end, the helix angle map (middle image) was also employed. 3 transmural sectors were defined evenly spanning the LV wall (right image). b) Bar graphs of DTI parameter [longitudinal diffusivity (λL), transverse diffusivity (λT), fractional anisotropy (FA), and mean diffusivity (MD)] zonal averages for the 6 rat hearts. The corresponding transmural sectors are color-encoded. Diffusivity unit is mm^2^/s. * denotes significance (*p*<0.05) of difference among the three transmural sectors.

Tensor data was fitted [4] and diagonalized on a pixel-by-pixel basis. Maps of fractional anisotropy (FA), and longitudinal (λL), transverse (λT) and mean (MD) diffusivities were computed. The equatorial short-axis slice with the largest area was analysed. 3 transmural sectors were defined evenly spanning the LV wall (Fig 1a). Zonal averages fοr all parameters were computed. To test the statistical significance of the differences among the zonal averages, one-way ANOVA was employed.

## Results

Distinct transmural gradients were observed (Fig 1b) in all measurements. Even though the exact mechanisms are still not perfectly understood, the measured extracellular diffusivity properties are, intuitively, sensitive to local cellular characteristics. Since the primary eigenvector is aligned to the cell long-axis, the revealed increase trend (*p*<0.05) of λL from the endo- to the epi-cardium is consistent with previous studies [5] on isolated LV myocytes that showed a similar trend in the cell length. The rises in measured extracellular λΤ (*p*<0.05) and MD (*p*<0.05) from the inner to the outer wall layers may be explained by the documented [6] reverse trend in the inversely proportional cell cross-sectional area. On similar grounds, the decrease in FA (*p*<0.05) from endo- to epi-cardium is in agreement with the manifested^5^ reverse trend in the cell length/width ratio.

## Conclusions

Significant transmural heterogeneity of preferential diffusion properties was observed in the normal rat myocardium. The results corroborate previous labor-intensive cellular morphometry studies on the same animal model. They may be used to improve our understanding of the heart functioning through dissecting critical underlying mechanisms.

## Funding

NIHR Cardiovascular BRU, Royal Brompton & Harefield NHS Trust and Imperial College London. NIH:R01 EB007219, the Director, SC, BER, Medical Sciences Division, U.S. DOE under Contract DE-AC02-05CH11231.
